# Quantum Discord for *d*⊗2 Systems

**DOI:** 10.1038/srep10262

**Published:** 2015-06-03

**Authors:** Zhihao Ma, Zhihua Chen, Felipe Fernandes Fanchini, Shao-Ming Fei

**Affiliations:** 1 Department of Mathematics, Shanghai Jiaotong University, Shanghai 200240, China; 2 Department of Physics and Astronomy, University College London, WC1E 6BT London, United Kingdom; 3 Department of Mathematics, College of Science, Zhejiang University of Technology, Hangzhou 310023, China; 4 Centre for Quantum Technologies, National University of Singapore, 117543 Singapore; 5 Departamento de Física, Faculdade de Ciências, Universidade Estadual Paulista; 6 School of Mathematical Sciences, Capital Normal University, Beijing 100048, China; 7 Max-Planck-Institute for Mathematics in the Sciences, 04103 Leipzig, Germany

## Abstract

We present an analytical solution for classical correlation, defined in terms of linear entropy, in an arbitrary 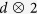 system when the second subsystem is measured. We show that the optimal measurements used in the maximization of the classical correlation in terms of linear entropy, when used to calculate the quantum discord in terms of von Neumann entropy, result in a tight upper bound for arbitrary 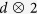 systems. This bound agrees with all known analytical results about quantum discord in terms of von Neumann entropy and, when comparing it with the numerical results for 10^6^ two-qubit random density matrices, we obtain an average deviation of order 10^−4^. Furthermore, our results give a way to calculate the quantum discord for arbitrary *n*-qubit GHZ and W states evolving under the action of the amplitude damping noisy channel.

Quantum entanglement plays important roles in many areas of quantum information processing, such as quantum teleportation and superdense coding^1^,^2^,^3^. Nevertheless, quantum entanglement is not the only form of quantum correlation that is useful for quantum information processing. Indeed, some separable states may also speed up certain quantum tasks, relative to their classical counterparts^4^,^5^,^6^,^7^, and many quantum tasks, such as quantum nonlocality^2^,^8^,^9^ and deterministic quantum computations with one qubit^10^, can be carried out with forms of quantum correlation other than quantum entanglement. One such quantum correlation, called quantum discord, has received a great deal of attention recently (see ref. 11 and references therein). Introduced by Ollivier and Zurek^12^ as the difference between the quantum mutual information and the maximal conditional mutual information obtained by local measurements^12^,^13^, quantum discord plays an important role in some quantum information processing^14^,^15^. Despite much effort by the scientific community, an analytical solution of quantum discord is still lacking even for two-qubit systems. Owing to the maximization involved in the calculation, there are only a few results on the analytical expression of quantum discord and only for very special states are exact solutions known. However, if instead of the von Neumann entropy one uses the linear entropy, the optimal measurements that maximize the conditional mutual information can be obtained analytically^16^. Here, we show that using these optimal measurements to determine the quantum discord in terms of the von Neumann entropy results in an excellent upper bound for the latter. Moreover, we show that this result gives a way to calculate the quantum discord for arbitrary *n*-qubit GHZ and W states, with each qubit subjected to the amplitude damping channel individually.

## Results

### Classical correlation under linear entropy

The usual quantum discord, in terms of von Neumann entropy, is defined as follows: let 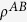 denote the density operator of a bipartite system composed of partitions *A* and *B*. Let 
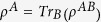
 and 
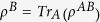
 be the reduced density operators of *A* and *B*, respectively. The quantum mutual information, which is the information-theoretic measure of the total correlation, is defined as 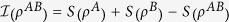, where 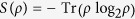 is the von Neumann entropy. Usually, the total correlation 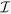 is split into the quantum part 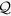 and the classical part 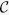, such that 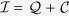. The classical correlation of a bipartite state 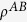 is defined as
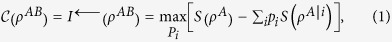
where the maximum is taken over all positive operator-valued measurements (POVM) 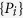 performed on subsystem *B*, satisfying 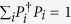, with probability of *i* as an outcome, 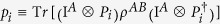 where 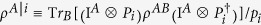 is the conditional state of system *A* associated with outcome *i*, where 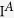 is the identity operator on subsystem *A*.

In this work, all POVM or projective measurements (PM) are taken on subsystem B. Finally, the quantum discord is defined as the difference between the total correlation and the classical correlation^12^,^13^:
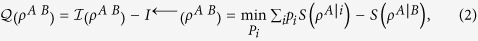
where 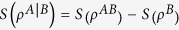 is the conditional entropy.

To calculate our tight upper bound to quantum discord, instead of the von Neumann entropy one uses the linear entropy. The linear entropy of a state 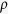 is given by:
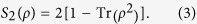


In terms of the linear entropy (3), one can correspondingly define the conditional linear entropy, 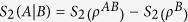, and the classical correlation^16^ is written as:
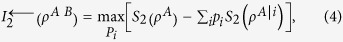
where the measurements run over all POVMs *P*_*i*_.

Although the classical correlation and, consequently, the quantum discord (2) is extremely difficult to compute in terms of von Neumann entropy, the classical correlation (4) expressed in terms of linear entropy can be calculated analytically. In what follows we present the analytical formula for an arbitrary 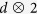 quantum systems.

A qudit state can be written as 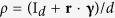, where 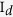 denotes the 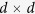 identity matrix, 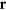 is a 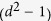-dimensional real vector, 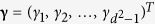 is the vector of generators of 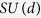 and 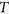 stands for transpose. Consider a bipartite system, composed of a 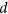-dimensional subsystem labeled *A* and a 2-dimensional subsystem labeled *B*. The bipartite state 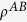 can be written as:
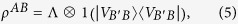
where 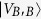 is the symmetric two-qubit purification of the reduced density operator 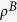 on an auxiliary qubit system 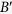 and 1 is the identity map on system *B*. Here, symmetric two-qubit purification means that the two reduced density matrices are equal, i.e. 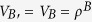, and 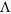 is a a completely positive trace-preserving map which maps a qubit state 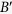 to the qudit state *A*. Let 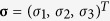 denote the vector of Pauli operators, **r** being a three-dimensional vector, 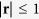. As a qubit state can generally be written as 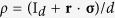, the map 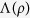 is of the form
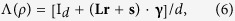
where **L** is a 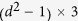 real matrix and **s** is a three-dimensional vector. **L** and **s** can be obtained from Eq. (5) and Eq. (6). Let 
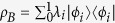
 be the spectral decomposition of 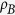. Then 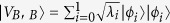 and 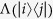, 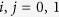, can be calculated by Eq. (5). Therefore one gets 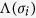, 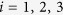, and the matrix 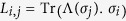. By the method used to calculate the classical correlation 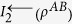 of two-qubit states^16^, we have:
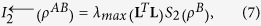
where 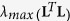 stands for the largest eigenvalue of the matrix 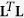. Eq. (7) gives the analytical formula for the classical correlation in terms of linear entropy for a general 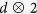 quantum state. Indeed, one only needs to find the eigenvalues of the matrix 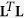.

Since, for a given state 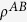, the reduced state 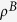, 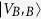 and the map 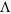 are fixed, the classical correlation can readily be computed in terms of linear entropy 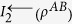. What concern us here are the optimal measurements that give rise to 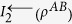. In fact, there is a one-to-one correspondence between all possible POVM measurements and all convex decompositions of 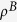 Ref. 17; namely, if 
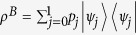
 is the pure state decomposition of 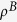, then the following are the corresponding POVMs:
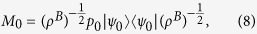

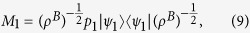
where 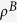 is full-ranked. Otherwise, we can find the inverse of 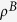 in its range projection and, from the optimal pure state decompositions of 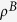, we can get the corresponding optimal POVMs. In Ref. 16, the authors have shown how to find the optimal decomposition of 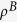. First write 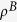 in its Bloch form: 
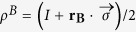
. Let 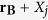 be the Bloch vector for the pure state decomposition 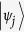 of 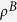, where 
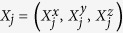
 and 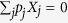, 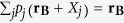. Hence, 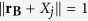.Then 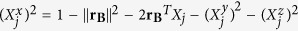. Without loss of generality, assume that 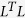 is diagonal with diagonal elements 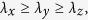 Eq. (7) becomes 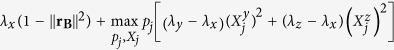, which gets the maximum value when 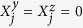. There are exactly two solutions of the equation 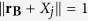. Hence the optimal decomposition of 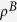 reads: 
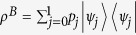
. From the two pure states in the optimal decomposition, we obtain the two optimal POVM measurement operators 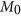 and 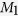.

It is well known that to maximize the classical correlation it is necessary to use the most general POVM quantum measurement. As it is much more complicated to find the maximum in (1) over all POVMs than over von Neumann measurements, almost all known analytical results are based on the latter. Indeed, only very few results are based on POVM^18^,^19^. Here, we show that for the case of a bipartite qudit-qubit state, the classical correlation based on linear entropy is maximized over projective measurements (see proof in the appendix). This leads to our first theorem:

**Theorem 1.** The classical correlation of a qudit-qubit state 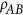 defined by running over all (arbitrary) POVM measurements is the same as the classical correlation defined by running over all projective measurements, i.e., 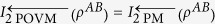.

### Quantum discord under von Neumann entropy

Theorem 1 implies that the optimal POVM in the classical correlation defined by Eq. (4) is in fact a projective measurement. This is very different from the case of classical correlation 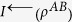 defined by von Neumann entropy, in which the classical correlation based on POVM could be larger than the one based on projective measurement^18^,^19^. This shows that, although von Neumann entropy and linear entropy have many properties in common, they behave quite differently in optimizing classical information. However, by using the optimal projective measurement for the classical correlation 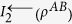 based on linear entropy, we can get a tight lower bound for the classical correlation based on von Neumann entropy, and hence a tight upper bound for the quantum discord based on von Neumann entropy. This leads us to our second theorem:

**Theorem 2.** The quantum discord based on von Neumann entropy has an upper bound:
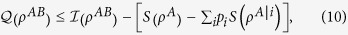
where 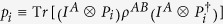 is the probability of the measurement outcome 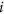, 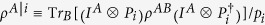 is the conditional state of system A when the measurement outcome is *i*, and 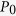 and 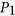 are the optimal projective measurement operators for 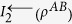 of a given 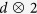 state 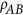.

In fact, there is a connection between discord and entanglement of formation (EOF): the classical correlation 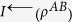 can be obtained from EOF by the Koashi Winter Relation^20^,
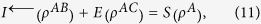
where 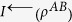 is the original classical correlation of 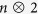 state 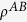, *E*(*ρ^AC^* ) is the EOF of state 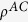, and 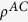 is the purification of 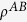. It is important to note that, from theorem 2, we can get an upper bound of EOF for arbitrary rank two 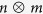 state 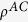.

Although the upper bound (10) of 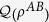 is given by the optimal measurement of 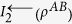, we show, by means of examples, that it is a surprisingly good estimate of 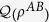.

*Example 1*. In Ref. 21 Luo presented the analytic formula for the quantum discord 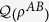 of the two-qubit Bell-diagonal state: 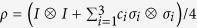. Let 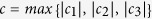. For this Bell-diagonal state, 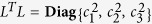 and 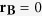. The two solutions of 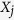 are 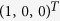 and 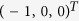 when 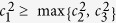: 
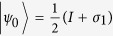
 and 
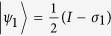
; 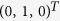 and 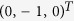 when 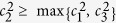: 
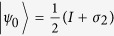
 and 
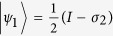
; 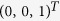 and 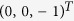 when 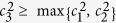: 
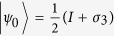
 and 
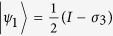
. It can be verified immediately that the optimal measurements for 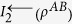 are given by 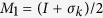 and 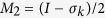, for 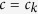 with 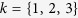. It can easily be checked that our upper bound (10) is exactly the same as the analytical results in ref. 21.

**Figure 1:**
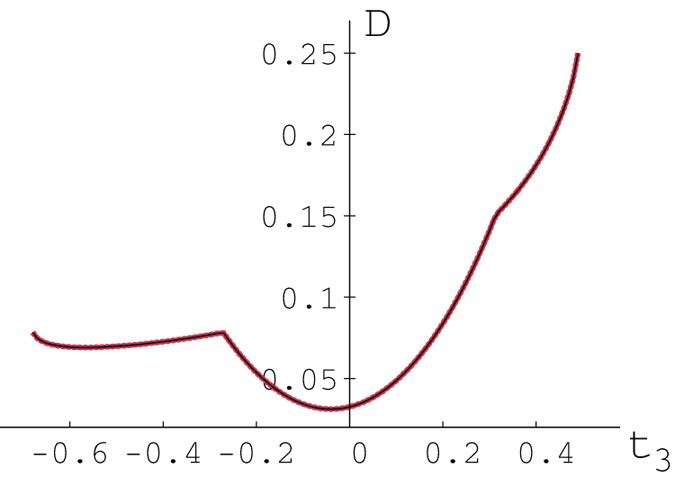
Quantum discord 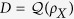 for 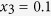, 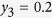, 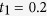, 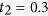. Here the results in 24, our numerical results and our upper bound in Eq. (10) agree with high precision.

*Example 2*. In ref. 22,23 the X-type two-qubit states are investigated: 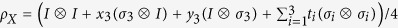, where 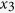, 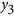, 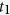, 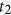 and 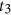 are defined such that 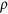 is a quantum state. It can easily be seen that our upper bound (10) agrees perfectly with the analytical results obtained in ref. 22 (see Fig. 1).

**Figure 2:**
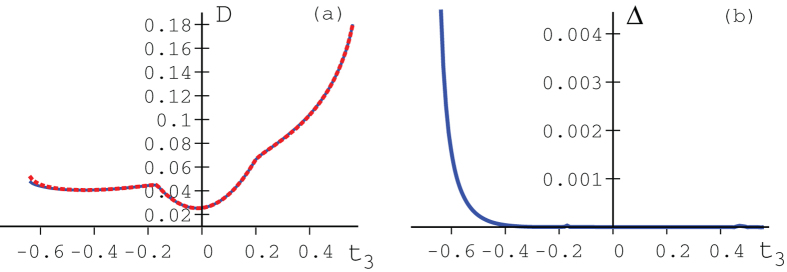
Figure (**a**) shows quantum discord 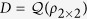. Solid blue line shows numerical results and the red dotted line our upper bound. Figure (**b**) shows the difference between the numerical results and our upper bound.

**Figure 3:**
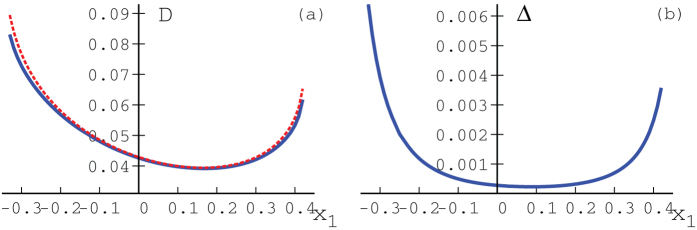
Figure (**a**) shows quantum discord 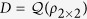. Solid blue line shows numerical results and the red dotted line our upper bound. Figure (**b**) shows the difference between the numerical results and our upper bound.

Now, let us consider the following general two-qubit states, 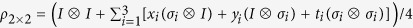, and compare our analytical upper bound with numerical results. Figure 2 gives the quantum discord 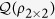, for 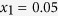, 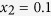, 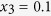, 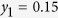, 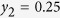, 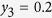, 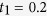 and 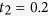 plotted against 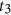, such that 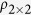 is a quantum state. Figure 3 shows the quantum discord 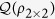 for 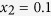, 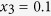, 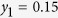, 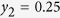, 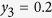, 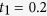, 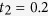 and 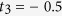, plotted against 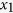, such that 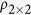 is a quantum state. It can be seen that our upper bound coincides very well with the numerical results.

**Figure 4:**
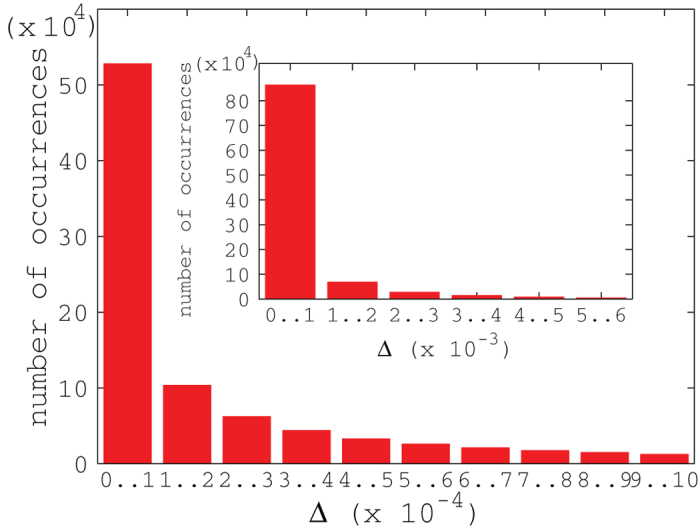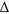 as a function of number of occurrences for a set of 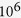 random 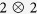 density matrices.

We have seen that the upper bound of quantum discord based on von Neumann entropy, obtained from the optimal measurements for the classical correlation based on linear entropy, is often exact. To test the precision of our upper bound generally, we calculated the difference between our analytical result and the numerical solution of quantum discord, for a set of 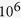 random density matrices of 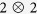. In Fig. 4, we plot the deviation 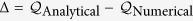 against the number of occurrences. It can be seen that more than half of the randomly generated density matrices results in a precision greater than 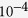, which demonstrates that our analytical result is a tight upper bound. Furthermore, in Fig. 4, we show that more than 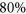 of the density matrices randomly generated lead to a precision greater than 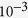. Indeed, the percentage of density matrices with a deviation 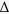 greater than 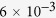 is less than 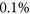. Here, in the horizontal coordinate of Fig. 4 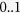 represents the interval from 0 to 1, and the same for 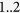, etc..

### Evolution of Quantum Discord under AD Channel

Now we consider the evolution of quantum discord for arbitrary 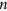-qubit GHZ and W states under an amplitude damping (AD) channel characterized by the Kraus operators 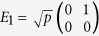 and 
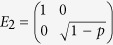
. We show that the related quantum discord based on von Neumann entropy can be analytically obtained from the upper bound given by Eq. (10).

**Figure 5:**
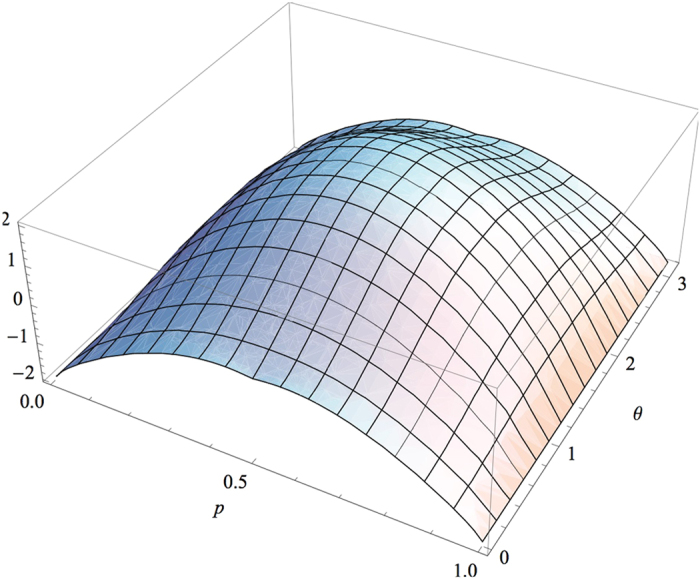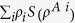 as a function of 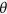 and p.

First let us consider 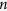-qubit GHZ states, with the first 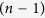 qubits subjected to AD channels individually. From Theorem 2, we get the optimal measurement operators 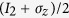 and 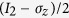 for classical correlation in terms of linear entropy, and the upper bound of quantum discord in terms of von Neumann entropy is then exact. Let 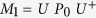 and 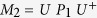 be the two measurement operators, where 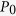 and 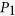 are the projective operators, 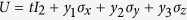 with 
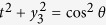
 and 
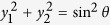
. Figure 5 shows that when 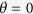 or 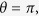 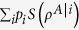 has the minimal value, which coincides with the optimal measurement operators 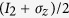 and 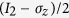 for classical correlation based on linear entropy.

For 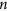-qubit W states with the first 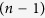 qubits subjected to individual AD channels, from Theorem 2 we have the optimal measurement operators 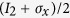 and 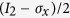 or 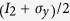 and 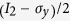. The upper bound of quantum discord obtained in terms of these measurement operators coincide with its lower bound in ref. 24. It follows that again we have the exact value of quantum discord (2).

Alternatively, if the last qubit of an 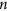-qubit W state is subjected to an AD channel, we have the optimal measurement operators 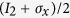 and 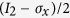 or 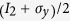 and 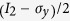, which also give rise to the exact value of discord (2).

## Conclusions

We have studied the quantum discord of qudit-qubit states. The analytical formula for classical correlation based on linear entropy has been explicitly derived, from which an analytical tight upper bound of quantum discord based on von Neumann entropy is obtained for arbitrary qudit-qubit states. The upper bound is found to be surprisingly good in the sense that it agrees very well with all known analytical results about quantum discord in terms of von Neumann entropy. Furthermore, for a set of 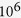 random density matrices, the maximum deviation found from the numerical solution was approximately 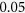 and the number of density matrices whose deviation was greater than 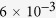 was less than 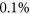 of the whole set. Our analytical results could be used to investigate the roles played by quantum discord in quantum information processing. For classical correlation in terms of linear entropy, it has also been shown that the result for a qudit-qubit state, defined by running over all two-operator POVM measurements, is equivalent to that defined by running over all projective measurements. Furthermore, our results can be applied to investigate the evolution of quantum discord for arbitrary 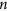-qubit GHZ and W states. Indeed, employing an important paradigmatic noisy channel, we present the quantum discord dynamics for the GHZ and W states when each qubit is subjected to independent amplitude damping channels.

## Additional Information

**How to cite this article**: Ma, Z. *et al*. Quantum Discord for d⊗_2_ Systems. *Sci. Rep.* **5**, 10262; doi: 10.1038/srep10262 (2015).
